# High density lipoprotein-associated proteins in non-obese women with and without polycystic ovary syndrome

**DOI:** 10.3389/fendo.2023.1117761

**Published:** 2023-04-26

**Authors:** Alexandra E. Butler, Abu Saleh Md Moin, Željko Reiner, Thozhukat Sathyapalan, Tannaz Jamialahmadi, Amirhossein Sahebkar, Stephen L. Atkin

**Affiliations:** ^1^ Research Department, Royal College of Surgeons in Ireland Bahrain, Adliya, Bahrain; ^2^ Department of Internal Medicine, University Hospital Center Zagreb, Zagreb, Croatia; ^3^ Academic Endocrinology, Diabetes and Metabolism, Hull York Medical School, Hull, United Kingdom; ^4^ Applied Biomedical Research Center, Mashhad University of Medical Sciences, Mashhad, Iran; ^5^ Biotechnology Research Center, Pharmaceutical Technology Institute, Mashhad University of Medical Sciences, Mashhad, Iran; ^6^ School of Medicine, The University of Western Australia, Perth, WA, Australia; ^7^ Department of Biotechnology, School of Pharmacy, Mashhad University of Medical Sciences, Mashhad, Iran

**Keywords:** polycystic ovary syndrome, lipids, HDL, LDL, dyslipidemia

## Abstract

**Introduction:**

Dyslipidemia frequently occurs in women with polycystic ovary syndrome (PCOS), but it is unclear whether dyslipidemia is due to obesity and insulin resistance (IR) or is inherent to PCOS. To address this, proteomic analysis of proteins important in lipid metabolism, particularly for high-density lipoprotein cholesterol (HDL-C), was performed in non-obese, non-insulin resistant PCOS women compared to matched controls.

**Methods:**

Weight and aged-matched non-obese subjects with PCOS (n=24) and without IR were compared with control women (n=24). 19 proteins were measured by Somalogic proteomic analysis: alpha-1-antichymotrypsin, alpha-1-antitrypsin, apolipoproteins A-1, B, D, E, E2, E3, E4, L1, M, clusterin, complement C3, hemopexin, heparin cofactor-II (HCFII), kininogen-1, serum amyloid A-1, amyloid beta A-4 and paraoxonase-1.

**Results:**

Women with PCOS had a higher free androgen index (FAI) (p<0.001) and anti-Mullerian hormone (AMH) (p<0.001), but IR and C-reactive protein (CRP), a marker of inflammation, did not differ from controls (p>0.05). The triglyceride:HDL-cholesterol ratio was elevated (p=0.03) in PCOS. Alpha-1-antitrypsin levels were lower (p<0.05) and complement C3 levels were higher (p=0.001) in PCOS. C3 correlated with body mass index (BMI) (r=0.59, p=0.001), IR (r=0.63, p=0.0005) and CRP (r=0.42, p=0.04) in women with PCOS, though no correlations of these parameters with alpha-1-antitrypsin were found. Total cholesterol, triglycerides, HDL-cholesterol, LDL-cholesterol and levels of the other 17 lipoprotein metabolism-associated proteins did not differ between the two groups (p>0.05). However, in PCOS, alpha-1-antichymotrypsin correlated negatively with BMI (r=-0.40, p<0.04) and HOMA-IR (r=-0.42, p<0.03), apoM correlated positively with CRP (r=0.36, p<0.04) and HCFII correlated negatively with BMI (r=-0.34, p<0.04).

**Conclusion:**

In PCOS subjects, when obesity, IR and inflammation confounders were absent, alpha-1-antitrypsin was lower and complement C3 was higher than in non-PCOS women, suggesting increased cardiovascular risk; however, subsequent obesity related IR/inflammation likely stimulates other HDL-associated protein abnormalities, thus increasing cardiovascular risk further.

## Introduction

Women with polycystic ovary syndrome (PCOS) have an increased prevalence of dyslipidemia (1), type 2 diabetes, metabolic syndrome, obesity, arterial hypertension, insulin resistance, endothelial dysfunction, hyperandrogenism and chronic low-grade inflammation; all these increase the risk of atherosclerotic cardiovascular disease (ASCVD) (2–4). Triglycerides, total cholesterol, LDL-cholesterol and atherogenic small dense LDL particles are higher while HDL-cholesterol is lower in obese versus normal weight PCOS subjects (5). HDL particles decrease cholesterol efflux capacity, which is reduced in PCOS thus increasing the risk of ASCVD (6). HDL particles have antiatherogenic effects, not only because of their cholesterol efflux capacity, but also because of anti-inflammatory, antithrombotic and anti-oxidative effects that regulate the expression of endothelial adhesion molecules; this prevents modified LDL particle generation and endothelial nitric oxide synthase stimulation (7). However, whilst obese women with PCOS have an increased prevalence of elevated triglycerides and lower HDL-cholesterol versus non-obese women without PCOS, the prevalence of hypercholesterolemia appears not to differ (8).

**Figure 1:**
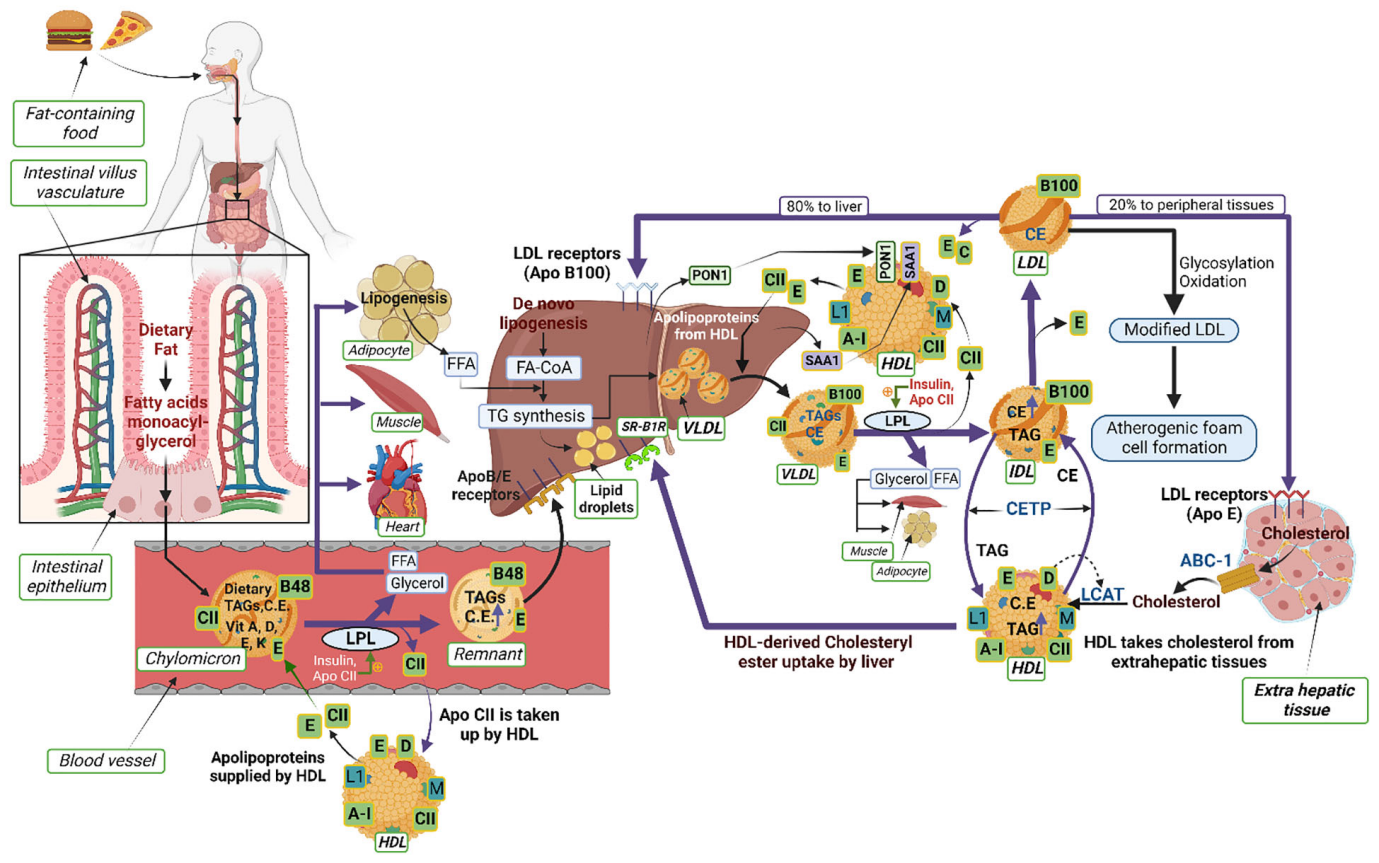
Schematic illustrating the interactions of proteins and lipids in the pathways of lipoprotein metabolism. Dietary fats (exogenous triglycerides) are carried by nascent chylomicrons synthesized by the intestine. Chylomicrons are assembled in the intestinal mucosal cells and carry dietary triacylglycerol (TAGs), cholesterol, fat soluble vitamins (vitamin A, D, E, K) and cholesteryl esters (CEs). TAGs account for close to 90% of the lipids in a chylomicron. Chylomicrons are hydrolyzed by an extracellular enzyme called lipoprotein lipase (LPL), which is anchored by heparan sulphate to the capillary wall of most tissues. LPL is activated by insulin and apolipoprotein CII (CII) on circulating chylomicrons; it hydrolyzes the TAGs to yield and free fatty acids (FFAs) and glycerol. In muscle (heart and skeletal muscle), fatty acids are oxidized for energy; in adipose tissue, they are re-esterized as TAGs for storage. Upon being hydrolyzed by LPL, a chylomicron is converted into a chylomicron remnant (which contains lipoproteins, Apo B-48, and Apo E). Chylomicron remnants (Apo B-48, Apo E) enter the liver through the low density lipoprotein (LDL) receptors (Apo B-100, E). Apo CII from chylomicrons is taken back by HDL. *De-novo* synthesis of fatty acids (endogenous triglycerides) in the liver are carried by nascent very low-density lipoprotein (VLDL) (containing lipoproteins Apo B-100, Apo CII and Apo E). Lipoprotein lipase hydrolyzes TAG to FFAs and the remnant of VLDL is called intermediate density lipoprotein IDL (containing Apo B-100, Apo E) which have lost triglyceride but are rich in cholesterol. The IDL and VLDL remnants (B-100, E) have 2 fates: most frequently, they lose Apo-E and are converted to LDL (B-100) and, less frequently, they are taken up by the liver *via* the LDL receptor. LDL (B-100) has 2 fates: 80% of LDL enters the liver through the LDL receptor (ApoB-100, E); 20% enters extrahepatic tissues through the LDL receptor (ApoE). LDL is also modified by glycosylation or oxidation and converted into atherogenic foam cells (in macrophages). HDL acts as a reservoir for different apoproteins and exchanges them with other lipoproteins; it provides Apo CII and E to nascent chylomicrons and VLDL to form chylomicrons (B-48, CII, E) and VLDL (B-100, CII, E). Besides being a reservoir for apoproteins, it serves the function of reverse cholesterol transport. HDL takes cholesterol from extrahepatic tissues through ATP binding cassette (ABC-1) transporter. Lectin-cholesterol acyltransferase (LCAT) in HDL (stimulated by A-1and Apo D) converts cholesterol to cholesteryl ester. Cholesteryl ester transport protein (CETP) mediates exchange of cholesteryl ester for triglyceride from HDL with the other lipoproteins. The liver reuptakes HDL-derived CEs through scavenger receptor class B type 1 (SR-B1). The other apoproteins of HDL are Apo L1 and Apo M. Two other HDL-associated proteins, paraoxonase-1 (PON1) and serum amyloid A1 (SAA1), are synthesized in the liver and later associated with the HDL moiety.

It appears that obesity is the main risk factor for insulin resistance (IR) in subjects with PCOS, though the IR elevation is multifactorial and the underlying mechanisms have not been fully elucidated. Although insulin receptor affinities are similar in women with or without PCOS, in PCOS the insulin binding in adipose tissue is decreased resulting in lower glucose uptake and IR. This may be due to a reduction of GLUT4 in subcutaneous adipose tissue in PCOS that could potentially promote IR (9, 10). The evidence suggests that the major mechanism underlying the abnormality in the insulin receptor leading to IR is a post-binding defect caused by increased serine phosphorylation and reduced tyrosine phosphorylation; this decreases activation of the phosphatidylinositol-3-kinase (PI3K) signaling pathway, the activator of glucose transport, by insulin (11). Pancreatic beta-cell dysfunction might be another reason for IR, since obese and overweight subjects with PCOS also have increased insulin secretion that causes excessive levels of proinsulin and results in IR (12). Some studies have indicated that HDL promotes pancreatic beta‐cell insulin secretion as well as modulating glucose uptake in peripheral tissues, thereby explaining the association between HDL, IR and obesity, including the obesity commonly present in women with PCOS (13). A strong link between HDL metabolism and glucose regulation has recently been reported (13, 14), though there are still many unresolved questions regarding the association between PCOS, IR and HDL particles. The associations between PCOS, inflammation and dyslipidemia are not clear. Specifically, PCOS is often characterized by low-grade inflammation; increased cholesterol in macrophages and other immune system-related cells involved in inflammation has been shown to promote inflammatory responses. However, inflammation also promotes cholesterol efflux pathways mediated by ABCA1, thereby mediating cholesterol efflux to lipid-free apoA1 and ABCG1; this, in turn, causes cholesterol efflux to nascent and mature HDL particles, events that may have beneficial effects by suppressing inflammatory responses (13–16) (**Figure 1**).

We hypothesized that the dyslipidemia seen in PCOS would have certain elements inherent to PCOS, but that obesity, IR and inflammation would likely be the major contributing factors. To address the inherent contribution of PCOS pathophysiology to dyslipidemia, non-obese, non-insulin resistant PCOS women without evidence of inflammation were compared with matched controls to determine how HDL-associated proteins participate in lipid and lipoprotein metabolism in PCOS. Subsequently, lipid metabolism-associated proteins were correlated to BMI, IR and inflammation to model their respective contributions with the development of obesity.

## Materials and methods

Plasma levels of proteins involved in lipoprotein metabolism were measured in non-obese, non-insulin resistant PCOS subjects (n=24) and weight and age matched control women without PCOS (n=24) attending the Hull IVF clinic (17). Demographic data for both the control and PCOS cohorts are shown in **Table 1**. As previously described (18), “All three diagnostic criteria of the Rotterdam consensus were used for the diagnosis of PCOS: (1) clinical and biochemical hyperandrogenemia requiring a Ferriman-Gallwey score of >8 and free androgen index of >4 respectively, (2) oligomenorrhea or amenorrhea and (3) polycystic ovaries seen on transvaginal ultrasound (19). Study participants had no other disease and were required to be medication-free for nine months before enrollment in the study. Fasting samples were collected on day 21 in normal controls and when all PCOS subjects were anovulatory (a minimum of six weeks after a period), at the time of a mock embryo transfer; all PCOS subjects were anovulatory with a progesterone less than 10pmol/l. Testing was performed to ensure that no patient had any of the following endocrine diseases: non-classical 21-hydroxylase deficiency, hyperprolactinemia, Cushing’s disease or an androgen-secreting tumor. All procedures were in accordance with the ethical standards of the Yorkshire and The Humber NRES ethical committee, UK, and the 1964 Helsinki declaration and its later amendments or comparable ethical standards. Written informed consent was obtained from all subjects.”

**Table 1:** Demographics, baseline, hormonal and metabolic parameters of the PCOS subjects and controls (mean ± SD), *p<0.05, **p<0.01.

	Control (n=24)	PCOS (n=24)
Age (years)	32.5 ± 4.1	31 ± 6.4
BMI (kg/m^2^)	24.8 ± 1.1	25.9 ± 1.8
Fasting glucose (nmol/L)	4.9 ± 0.4	4.7 ± 0.8
HbA1C (mmol/mol)	30.9 ± 6.5	31.8 ± 3.0
HOMA-IR	1.8 ± 1.0	1.9 ± 1.6
SHBG (nmol/L)	104.2 ± 80.3	71.7 ± 62.2
Free androgen index (FAI)	1.3 ± 0.5	4.1 ± 2.9**
CRP (mg L^-1^)	2.3 ± 2.3	2.8 ± 2.6
AMH (ng/ml)	24 ± 13	57 ± 14**
Cholesterol (mmol/L)	4.8 ± 0.8	4.7 ± 1.0
Triglycerides (mmol/L)	1.0 ± 0.5	1.3 ± 0.7
HDL (mmol/L)	1.7 ± 0.4	1.5 ± 0.4
LDL (mmol/L)	2.7 ± 0.6	2.6 ± 0.8
TG : HDL ratio	0.6 ± 0.3	0.9 ± 0.6*

BMI, Body Mass Index; HbA1c- glycated hemoglobin; HOMA-IR, Homeostasis model of assessment – insulin resistance; CRP, C reactive protein; SHBG, sex hormone binding globulin; AMH, Anti-Müllerian hormone; HDL, high density lipoproteins; LDL, low density lipoproteins; TG, triglycerides.

Lipoprotein metabolism-related plasma proteins were measured by Slow Off-rate Modified Aptamer (SOMA)-scan (Somalogic, Boulder, CO, USA) (20). “Normalization of raw intensities, hybridization, median signal and calibration signal were performed based on the standard samples included on each plate,” as has previously been described (21).

Version 3.1 of the SOMAscan Assay was used, targeting those proteins specifically involved in lipoprotein metabolism in the SOMAscan panel. These 19 proteins were alpha-1-antichymotrypsin, alpha-1-antitrypsin, apolipoproteins A-1, B, D, E, E2, E3, E4, L1 and M, clusterin, complement C3, hemopexin, heparin cofactor II, kininogen-1, serum amyloid A-1, amyloid beta A-4 and paraoxonase-1.

## Statistics

As described previously (18), “a power analysis (nQuery version 9, Statsol USA) was performed for the complement C3 protein previously reported to be different in women with PCOS (22). For 80% power and alpha of 0.05 with a common standard deviation (SD) of 0.37, the number of subjects required was 23. Trends in the data were visually inspected and statistically evaluated for normality. The Student’s t-test was applied on Gaussian distributed data while the Mann-Whitney (non-parametric) test was applied on non-Gaussian data as determined by the Kolmogorov-Smirnov test.” Correlation analyses with simple linear regression were performed with the proteins of interest and demographic and biochemical parameters of interest. All analyses were performed using Graphpad Prism version 9.4.1 (San Diego, CA, USA).

## Results

Baseline data for the 24 PCOS subjects and 24 control subjects is shown in **Table 1**. Subjects with PCOS had an elevated free androgen index and an elevated level of anti-Mullerian hormone. PCOS subjects were non-obese, non-insulin resistant and well matched for age and BMI compared to controls. The two cohorts did not differ in CRP levels (as a marker of inflammation). Total cholesterol, triglycerides (TG), high-density lipoprotein cholesterol (HDL-C) and low-density lipoprotein cholesterol (LDL-C) levels were similar in both cohorts, although TG : HDL-C ratio was elevated (p=0.03) in the women with PCOS.

### Levels of proteins involved in lipoprotein metabolism in PCOS

The results of the Somascan analysis of lipid metabolism-related proteins are shown in **Table 2** for the women with PCOS and control women.

**Table 2:** Levels of proteins involved in lipid metabolism in subjects with polycystic ovary syndrome (PCOS) fulfilling all three diagnostic criteria versus controls.

	PCOS	Control	p value
Alpha-1-antichymotrypsin	248189 (28942)	228269 (47184)	0.06
Alpha-1-antitrypsin	892 (268)	1371 (968)	0.01
Apolipoprotein A-I	12392 (2448)	12344 (2248)	0.94
Apolipoprotein B	7224 (4806)	5871 (2640)	0.19
Apolipoprotein D	2586 (497)	2457 (630)	0.39
Apolipoprotein E	26588 (13321)	25296 (11517)	0.69
Apolipoprotein E2	221851 (56020)	214964 (67686)	0.68
Apolipoprotein E3	157453 (59480)	148321 (59451)	0.56
Apolipoprotein E4	173799 (63642)	170531 (57851)	0.84
Apolipoprotein L1	38171 (10597)	38852 (11467)	0.82
Apolipoprotein M	12971 (4684)	14029 (5181)	0.42
Clusterin	797 (105)	849 (166)	0.16
Complement C3	65878 (26872)	45742 (18189)	0.001
Hemopexin	1143 (471)	1014 (469)	0.30
Heparin cofactor II	3767 (817)	3866 (1000)	0.68
Kininogen-1	26906 (6281)	28203 (7893)	0.49
Serum amyloid A-1	1211 (1150)	1585 (2774)	0.51
Amyloid beta A4	28300 (22332)	31246 (19569)	0.60
Paraoxonase 1	126 (23)	130 (47)	0.73

Data presented as Mean ± 1 Standard Deviation of Relative Fluorescent Units (RFU).

**Figure 2:**
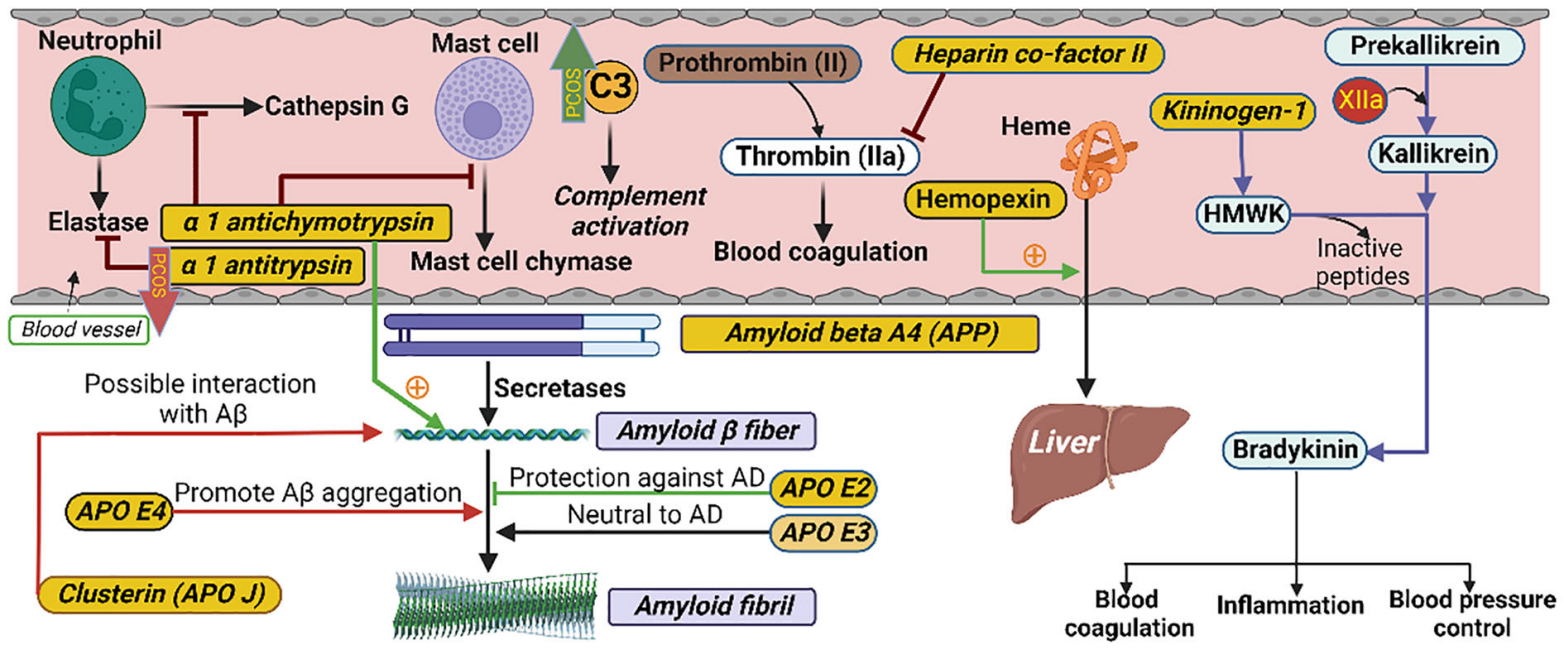
Schematic illustrating the interactions of proteins in their respective biological pathways measured in the current study. α-1 antichymotrypsin (ACT) is a plasma serine protease inhibitor. Its main physiological target is neutrophil cathepsin G as well as mast cell chymase. ACT is an integral component of the amyloid deposits in Alzheimer’s disease (AD) and has been shown to catalyze amyloid beta (Aβ) polymerization. Alpha 1 antitrypsin (A1AT) is a serum proteinase inhibitor, especially the neutrophil elastase. Heparin co-factor II is a coagulation factor that inhibits factor IIa (thrombin) in the blood coagulation system. Hemopexin is a plasma glycoprotein with the highest binding affinity to heme. Its main function is scavenging (from blood to liver) the heme released or lost by the turnover of heme proteins such as hemoglobin. Kininogen-1 (in humans encoded by the KNG1 gene) is a α-2-thiol proteinase inhibitor and a constituent of the blood coagulation system as well as the kinin-kallikrein system. The KNG1 gene undergoes alternative splicing to generate high molecular-weight kininogen (HMWK) and low molecular-weight kininogen. HMWK, in turn, is cleaved by the enzyme kallikrein (synthesized by pre-kallikrein with the help of coagulation factor XIIa) to produce bradykinin. Bradykinin is a potent endothelium-derived vasodilator and is involved in different biological processes including blood coagulation, inflammation and blood pressure control. Amyloid beta A4, also known as amyloid precursor protein (APP), functions as a cell surface receptor and transmembrane precursor protein which is cleaved by secretases to form amyloid beta (Aβ) fibers. Human APOE lipoprotein isoforms, APOE2, APOE3 and APOE4, are involved in the pathobiology of AD. While APOE2 has a protective effect against amyloid fibril formation, APOE3 has no effect in amyloid fibril generation. APOE4, which promotes Aβ aggregation, constitutes the highest genetic risk factor for AD. APOJ (also known as clusterin) has also been found to be positively interacted with Aβ. C3 is the most important and abundant protein in the complement system. The level of C3 was higher and the level of alpha 1 antitrypsin was lower in PCOS as indicated by the green upward and red downward arrows, respectively.

Alpha-1-antitrypsin levels were lower (892 ± 268 vs 1371 ± 968 RFU, PCOS vs control, p=0.01) in non-obese, non-insulin resistant women with PCOS when compared with controls. Complement C3 levels were higher in the PCOS cohort (65878 ± 26872 RFU, PCOS vs control, p=0.001). The levels of the other 17 proteins associated with lipoprotein metabolism were not different between the two cohorts, although there was a trend for elevation of alpha-1-antichymotrypsin in women with PCOS (p=0.06) (**Table 2**; **Figure 2**).

### Correlation analyses

For the two proteins that differed in women with PCOS versus control women (complement C3 and alpha-1-antitrypsin), correlations with age, BMI, insulin resistance (HOMA-IR), testosterone and circulating levels of TG, cholesterol, HDL-C, LDL-C and CRP were performed.

**Figure 3:**
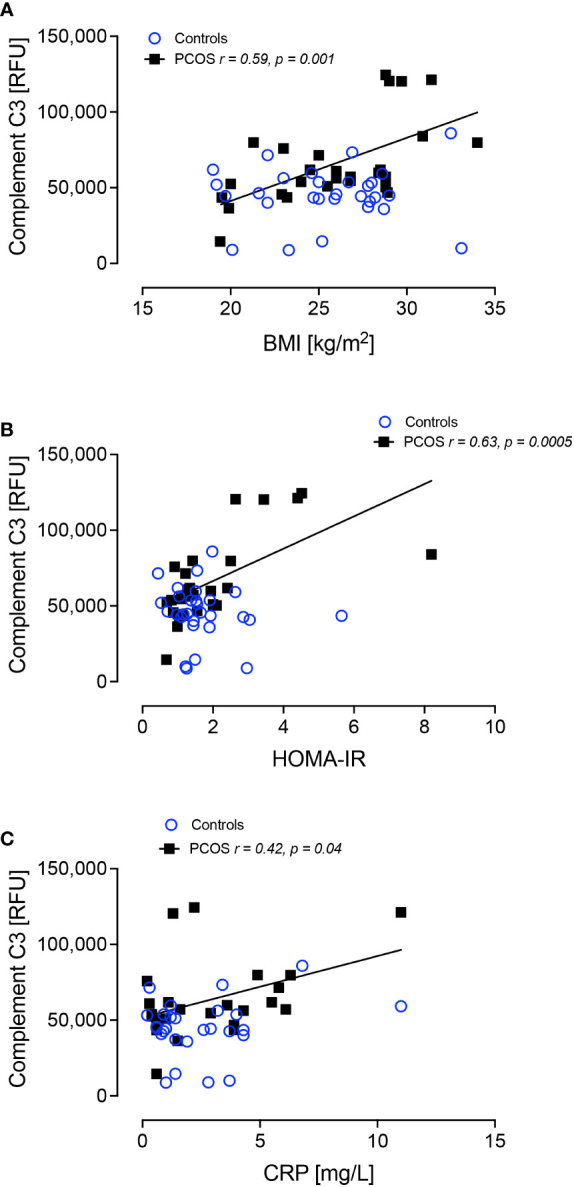
Complement C3 correlated significantly with BMI in PCOS **(A)**, HOMA-IR in PCOS **(B)** and C-reactive protein in PCOS **(C)**.

Complement C3 positively correlated with BMI (r=0.59, p=0.001), HOMA-IR (r=0.63, p=0.0005) and CRP (r=0.42, p=0.04) only in women with PCOS (**Figure 3**). Alpha-1-antitrypsin did not correlate with BMI, HOMA-IR or CRP (p>0.05)

**Figure 4:**
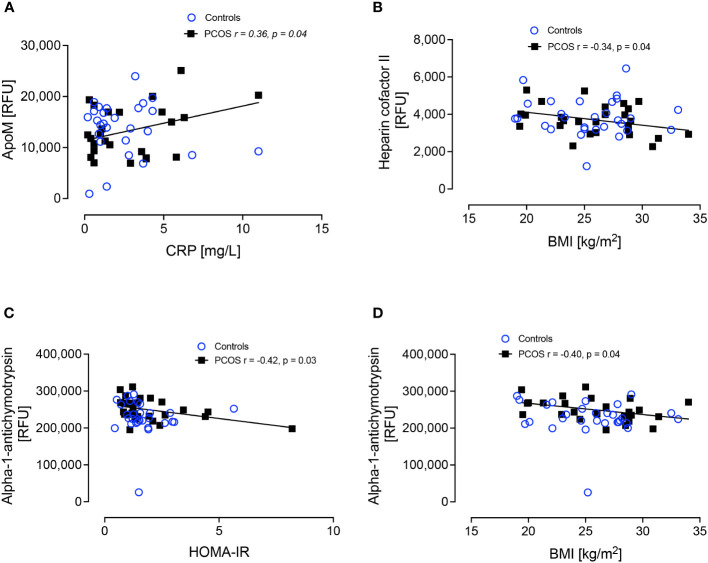
Correlations of ApoM and CRP **(A)**, Heparin cofactor II and BMI **(B)**, alpha-1-antichymotrypsin and HOMA-IR **(C)** and alpha-1-antichymotrypsin and BMI **(D)**.

Since complement C3 correlated with BMI in the women with PCOS despite them having a normal BMI, further correlations with BMI, HOMA-IR and CRP were performed for proteins alpha-1-antichymotrypsin, apolipoproteins A-1, B, D, E, E2, E3, E4, L1 and M, clusterin, hemopexin, heparin cofactor II, kininogen-1, serum amyloid A-1, amyloid beta A-4 and paraoxonase-1. ApoM correlated positively with CRP (r=0.36, p<0.04), heparin cofactor-II correlated negatively with BMI (r=-0.34, p<0.04) and alpha-1-antichymotrypsin correlated negatively with BMI (r=-0.40, p<0.04) and with HOMA-IR (r=-0.42, p<0.03), but these correlations were found only in the women with PCOS (**Figure 4**).

## Discussion

This study shows that, when obesity, insulin resistance and inflammation are removed as confounding factors in women with PCOS, there was an association between dyslipidemia and alpha-1-antitrypsin and complement C3, suggesting there are inherent differences in women with PCOS compared to control women. It has been suggested that alpha-1-antitypsin might be a pro-inflammatory protein, thus compromising the protective role of HDL particles when carried on their surface and causing their dysfunctionality (21). Upregulation of alpha-1-antitrypsin on HDL enhances the anti-inflammatory functionality of these particles and alpha-1-antitrypsin-enriched HDL particles have increased anti-inflammatory activity. Therefore, lower levels of alpha-1-antitrypsin, as were found in the women with PCOS in this study, might compromise the antiatherogenic activity of HDL (23, 24). Alpha-1-antitrypsin did not correlate with BMI, IR or inflammation, suggesting that the difference seen here between women with PCOS and controls is inherent to the PCOS disorder. To date, there is only one study analyzing alpha-1-antytrypsin in PCOS which reported that alpha-1-antitrypsin was upregulated in the follicular fluid of PCOS subjects (25).

The finding that complement C3 levels were elevated in PCOS subjects with normal weight is important since increased levels of complement C3 have been associated with increased risk of atherosclerosis (26). HDL subspecies containing complement C3 are associated with higher CHD risk versus HDL subspecies lacking complement C3 (27, 28). Complement C3 is associated with an adverse lipoprotein profile distinguished by an increase in triglyceride-enriched lipoproteins and a reduction in large HDL particles (29); reduced levels of large HDL particles has been associated with increased CHD risk (30). Although the subjects with PCOS in this study had a normal BMI, were not insulin resistant and had no evidence of inflammation, BMI, HOMA-IR and CRP positively correlated with C3. This is particularly intriguing since it may suggest that even in women with PCOS who have normal body weight, are not insulin resistant and do not have increased inflammatory markers, they may still have an elevated CVD risk versus those without PCOS and, of critical importance, weight gain in the setting of PCOS may have a disproportionately deleterious effect.

Most of the HDL-associated proteins were normal and did not differ between the non-obese PCOS subjects and control women. However, modeling the HDL-associated protein parameters with BMI, IR and inflammation (CRP) showed associations which were found only in the women with PCOS. What these correlations with BMI indicate are the detrimental changes that are predicted to occur with the development of overt obesity. Alpha-1-antichymotrypsin, an inflammatory biomarker (31), correlated negatively with both BMI and HOMA-IR. Although there is no data in the literature on its modulation by either of these parameters, by analogy with alpha-1-antitrypsin, its reduction with increased BMI and IR may be related to a reduction of its protective properties. Heparin cofactor-II negatively correlated with BMI. HCFII has been reported to be raised in obese women with PCOS and, since HCFII inhibits thrombin action, its decrease could be considered as an increased cardiovascular risk factor (32). ApoM correlated with CRP. This is important since apoM is a modulator of sphingosine 1-phosphate (S1P) and is considered to be responsible for the pleiotropic effects of HDL, such as reverse cholesterol transport, anti-inflammatory and anti-oxidant activity as well as mediating pre-β-HDL formation; the metabolism of apoM-containing lipoproteins is the major factor affecting plasma S1P levels (33). Recently, it was shown that S1P stimulates cholesterol efflux *via* the ABCA1/ApoA-I axis, suggesting the role of both S1P and the ABCA/ApoA-I axis in cholesterol efflux from macrophages (34). ApoM is therefore considered to be one of the important HDL particle-bound cardioprotective apolipoproteins and is negatively associated with ASCVD risk (35). When HDL/apoM-bound S1P binds to endothelial cell S1P1/S1P3 receptors, the effect is potently antiatherogenic. However, by contrast, when HDL/apoM-bound S1P acts upon S1P2 receptors, the effect may be proatherogenic (36, 37). ApoM did not correlate with BMI suggesting that the decreased apoM seen in obese subjects may be result of an indirect effect of increased BMI. Nevertheless, apoM strongly correlates with CRP (38). Systemic inflammation impacts HDL particle apolipoprotein and lipid composition, causing changes in their function and impairing their vasculo-protective effects (39).

Modeling the predicted changes as a response to increasing BMI, IR and inflammation suggests the following: a significantly increase in C3, with a decrease in heparin cofactor-II, apoM and alpha-1-antichymotrypsin; however, these changes may only be seen in women with PCOS and not in obese control women, indicating an increased cardiovascular risk for obese women with PCOS.

HDL-C and the main apolipoprotein of HDL particles, ApoA1, are significantly lower in obese women with PCOS when compared with controls (40), but they did not differ here in non-obese women with PCOS and neither were they associated with BMI, IR or inflammation. A number of studies have shown that obese subjects with PCOS had a more atherogenic lipoprotein profile consisting of reduced numbers of HDL and increased numbers of LDL particles, increased numbers of very low-density lipoprotein particles (VLDL) and reduced numbers of LDL and HDL particles when compared with women of similar age and BMI but without PCOS (41, 42). This was shown to be particularly true for Hispanic subjects compared with non-Hispanic white women (43) suggesting that there are ethnic differences within the PCOS spectrum in terms of cardiovascular risk. As the lipid levels did not differ between PCOS and control women, why should there be correlations with proteins that are associated with an adverse cardiovascular risk in women with PCOS but not in normal control women? Speculatively, this may result from the composition of the lipids noted above that have been shown to be different between PCOS and controls, with PCOS subjects reported to have increased levels of atherogenic lipids (41, 42).

The strength of this study was that this was a non-obese PCOS cohort that did not have either inflammation or increased IR. This is the only study design that could adequately address the question, as BMI is so highly correlated with PCOS. As such, if an obese population was studied, then regression adjustment for BMI would over-adjust the model, removing some of the PCOS effects. However, to confirm the postulated changes in the proteins, an obese PCOS cohort is needed. Given that cholesterol levels do vary through the menstrual cycle, with total cholesterol levels increasing as estrogen levels increase and HDL-C maybe being highest at ovulation (44), the fasting samples in this study were taken at the same time (at the time of mock embryo transfer, that was day 21 in the normal controls and when the PCOS subjects were anovulatory). Whilst optimized for the specific time of the cycle when cholesterol levels may be at their lowest, this does mean that comparator studies in the follicular phase of the cycle should be done to see if the correlations reported here span the menstrual cycle or are menstrual cycle specific. This study has several limitations. The doubts concerning a report such as this is that our negative findings could be a type 2 statistical error caused by inadequate sample size. However, the initial power analysis based on complement C3 offers reassurance that this was properly addressed. The SOMAscan platform results are reported in Relative Fluorescent Units (RFU) and there is no conversion formula from RFU to protein concentration. Since all women were Caucasian, it may not be possible to generalize these results to other ethnic groups. In future studies, the role of the different PCOS phenotypes (45) based upon the Rotterdam criteria for diagnosis, needs to be addressed.

## Conclusion

This study showed that when obesity, IR and inflammation parameters were excluded as confounding factors in subjects with PCOS, HDL-associated alpha-1-antitrypsin and complement C3 were still abnormal. However, the associations found suggest that subsequent obesity development in women with PCOS, with increased IR and inflammation, would likely result in significantly increased C3 and decreased heparin cofactor-II, apoM, alpha-1-antichymotrypsin and alpha-1-antichymotrypsin, thus further increasing their cardiovascular risk.

## Ethics approval and consent to participate

All procedures performed in studies involving human participants were in accordance with the ethical standards of the Yorkshire and The Humber NRES ethical committee, UK, and with the 1964 Helsinki declaration and its later amendments or comparable ethical standards. Written informed consent was obtained from all subjects.

## Data availability statement

The raw data supporting the conclusions of this article will be made available by the authors, without undue reservation.

## Ethics statement

The studies involving human participants were reviewed and approved by the Yorkshire and The Humber NRES ethical committee, UK. The patients/participants provided their written informed consent to participate in this study.

## Author contributions

AB, AS and SA conceptualized the study. AB and AM analyzed the data and wrote the manuscript. TS supervised clinical studies and edited the manuscript. ZR, TJ and AS contributed to data interpretation and writing the manuscript. SA contributed to data interpretation and writing the manuscript. All authors reviewed and approved the final version of the manuscript. AB is the guarantor of this work.
